# Calcium and Excitation-Contraction Coupling in the Heart

**DOI:** 10.1161/CIRCRESAHA.117.310230

**Published:** 2017-07-06

**Authors:** David A. Eisner, Jessica L. Caldwell, Kornél Kistamás, Andrew W. Trafford

**Affiliations:** From the Unit of Cardiac Physiology, Division of Cardiovascular Sciences, Manchester Academic Health Sciences Centre, University of Manchester, United Kingdom.

**Keywords:** calcium, cytoplasm, mitochondria, ryanodine receptor calcium release channel, sarcoplasmic reticulum

## Abstract

Cardiac contractility is regulated by changes in intracellular Ca concentration ([Ca^2+^]_i_). Normal function requires that [Ca^2+^]_i_ be sufficiently high in systole and low in diastole. Much of the Ca needed for contraction comes from the sarcoplasmic reticulum and is released by the process of calcium-induced calcium release. The factors that regulate and fine-tune the initiation and termination of release are reviewed. The precise control of intracellular Ca cycling depends on the relationships between the various channels and pumps that are involved. We consider 2 aspects: (1) structural coupling: the transporters are organized within the dyad, linking the transverse tubule and sarcoplasmic reticulum and ensuring close proximity of Ca entry to sites of release. (2) Functional coupling: where the fluxes across all membranes must be balanced such that, in the steady state, Ca influx equals Ca efflux on every beat. The remainder of the review considers specific aspects of Ca signaling, including the role of Ca buffers, mitochondria, Ca leak, and regulation of diastolic [Ca^2+^]_i_.

The process of excitation–contraction (E–C) coupling links the electric excitation of the surface membrane (action potential) to contraction. Since the initial measurements in cardiac muscle,^1,2^ an enormous amount of work has shown the underlying changes of cytoplasmic calcium concentration ([Ca^2+^]_i_).^3,4^ Ca binds to troponin resulting in sliding of the thick and thin filaments, cell shortening, and thence the development of pressure within the ventricle and ejection of blood. Force, therefore, depends on the amount of Ca bound to troponin. This will be a function of both the magnitude and duration of the rise of [Ca^2+^]_i_. It will also depend on the strength of Ca binding, a factor that can be altered genetically,^5^ is controlled by factors such as phosphorylation^6^ and may form the basis of therapeutic interventions.^7^ Nevertheless, the major factor that regulates contraction is the level of intracellular Ca. As well as focusing on the increase of [Ca^2+^]_i_ during systole, it is important to remember that proper cardiac function requires that force and [Ca^2+^]_i_ relax quickly to low enough levels such that the heart can refill with blood. Therefore, both diastolic and systolic [Ca^2+^]_i_ must be tightly regulated; this regulation is the subject of the current article.

The events that occur in E–C coupling are now well established (Figure 1). The process depends not only on a combination of the properties of Ca channels and transporters but, equally importantly, also on their precise locations and spatial arrangement. The depolarization produced by the action potential opens L-type Ca channels situated in the surface membrane and transverse tubules. The resulting entry of a small amount of Ca results in a large increase of [Ca^2+^]_i_ in the dyadic space (the region bounded by the t-tubule and sarcoplasmic reticulum [SR]). This increase of [Ca^2+^]_i_ makes the SR Ca release channels (ryanodine receptors [RyR]) open thereby releasing a much larger amount of Ca from the SR in a process termed calcium-induced calcium release. The magnitude of the rise of [Ca^2+^]_i_ depends not only on the structures mentioned above but also on Ca binding to buffers and uptake into organelles including mitochondria. For relaxation to occur, Ca must be removed from the cytoplasm. This requires that the RyRs close and then that Ca is pumped (1) back into the SR, by the SERCA (SR Ca-ATPase) and (2) out of the cell, largely by the sodium–calcium exchange (NCX).

**Figure 1.**
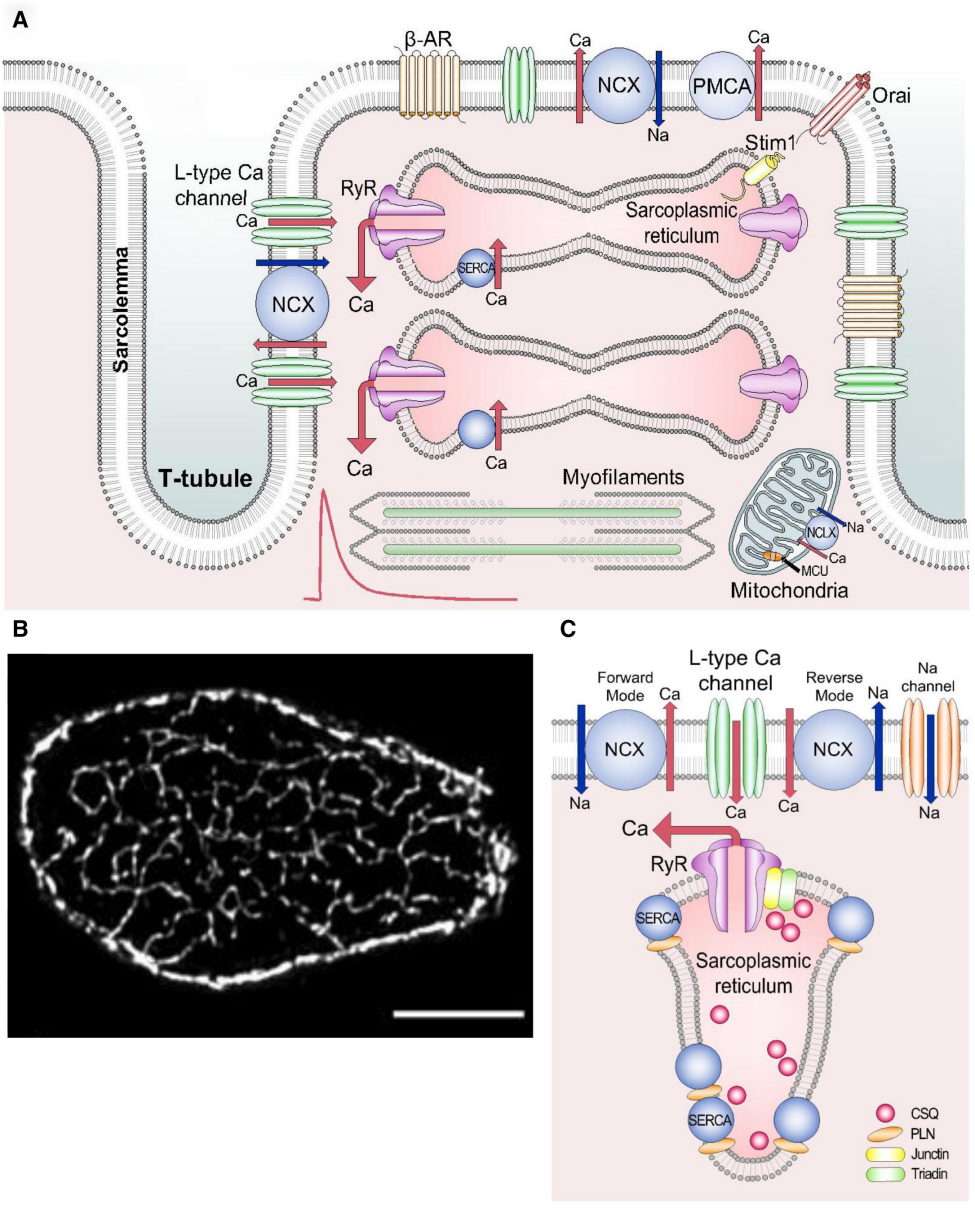
**Structures involved in Ca cycling. A**, Schematic diagram. This shows surface membrane, transverse tubule, sarcoplasmic reticulum (SR), and mitochondria, as well as the various channels and transporters mentioned in the text. **B**, High-resolution transverse section of a ventricular myocyte showing t-tubule network. Reprinted from Jayasinghe et al^39^ with permission of the publisher. Copyright ©2009, Biophysical Society. **C**, Cartoon of dyad emphasizing the major proteins involved in Ca cycling. B-AR indicates beta adrenoceptor; MCU, mitochondrial Ca uniporter; NCX, sodium–calcium exchange; NCLX, mitochondrial Na–Ca exchange; PMCA, plasma membrane Ca-ATPase; RyR, ryanodine receptor; and SERCA, sarco/endoplasmic reticulum Ca-ATPase.

The remainder of this review is in 3 sections. In the first, we discuss recent studies on the spatial organization of the structures responsible for calcium cycling. The second addresses the general principles that determine how the amplitude of the Ca transient is controlled, with particular reference to understanding the importance of Ca flux balance. In the final section, we consider specific steps in Ca signaling.

## Structural Considerations: The Dyad

Transverse (t-) tubules are 150- to 300-nm-wide^8,9^ deep invaginations of the surface sarcolemma occurring at the junction of each sarcomere (z-line). They are observed in ventricular myocytes from all mammalian species studied. E–C coupling depends on the close association between the SR network and t-tubule membranes.^10,11^ The junctional SR makes close contact with the t-tubule membrane so that RyRs on the SR are very closely apposed (≈15 nm^12^) to L-type Ca^2+^ channels on the t-tubule thus forming the cardiac dyad that is fundamental to the processes initiating the systolic Ca^2+^ transient.

When viewed end on, t-tubules are seen to radiate throughout the ventricular cell (Figure 1B). The close association between the t-tubule and SR ensures the synchronous rise of [Ca^2+^]_i_ during systole.^13^ Indeed, chemical detubulation in ventricular myocytes with formamide results in a markedly heterogenous Ca^2+^ transient commencing at the surface sarcolemma with a slowly propagating wave of Ca^2+^ release traveling to the cell center.^14^ This has similar spatial properties to the systolic Ca^2+^ transient in those atrial cells lacking t-tubules.^15,16^ (Although outside the scope of the present article, it should be noted that atrial myocytes from larger mammalian species have a well-developed ventricular-like t-tubule network^17–19^).

The cardiac dyad is a specialized signaling nexus concerned primarily with the initiation of cardiac contraction. Classically, it consists of clusters of L-type Ca^2+^ channels on the sarcolemma closely apposed (≈15 nm) across the dyadic cleft to clusters of RyRs on the SR membrane. In addition to these basic requirements for excitation–contraction coupling, the cardiac dyad may also be considered as containing additional structures that may contribute to or modulate Ca^2+^ release from the SR during systole (Figure 1). Of these, the most extensively studied is NCX that has been argued via its reverse-mode action to contribute to Ca^2+^ influx early during the action potential.^20^ However, assuming dyadic and cytosolic intracellular Na^+^ are similar during diastole (5–10 mmol/L,^21^) such reverse-mode NCX is thermodynamically limited leading to the suggestion that Na^+^ entry via voltage-gated Na^+^ channels (*I*_Na_) may raise dyadic Na^+^ sufficiently early during the action potential to facilitate effective reverse-mode NCX. Indeed, Leblanc and Hulme^22^ first demonstrated the modulating effect of *I*_Na_ on Ca^2+^ release from the SR. Subsequent experiments suggested that a subpopulation of neuronal Na^+^ channels are localized to the t-tubule and thence dyadic environ^23–25^; however, Brette et al^26^ also concluded that although neuronal Na^+^ channels were concentrated on the t-tubule, they were not required for cardiac excitation–contraction coupling.

Major changes occur in dyadic structure in heart failure with a reduction in the number of t-tubules in the ventricle^27^and, in the atrium, the loss of virtually all.^18^ T-tubule loss and the consequent loss of tight coupling between L-type Ca^2+^ channels and RyRs result in the so-called orphaned RyRs and a reduction in the synchronicity and amplitude of the Ca^2+^ transient.^28,29^

Until recently, it was unclear how many RyRs make up a cluster. The advent of super-resolution imaging methods has provided estimates for the number of RyRs in each cluster (dyad) from ≈14 in peripheral couplings to ≈100 in intracellular sites.^10,30,31^ The significance of RyR cluster size and cluster homogeneity currently lacks direct experimental evidence. However, simulation studies have related the number of RyRs per cluster (ie, cluster size) and the uniformity of the cluster (ie, the presence of gaps between individual RyRs) to the properties of (1) Ca^2+^ sparks^32,33^ and (2) the synchronicity of systolic Ca^2+^.^34^ In particular, larger and more uniformly packed clusters were modeled to be more likely to give rise to a Ca^2+^ spark, and larger clusters uniformly distributed throughout the cell gave rise to a more synchronous rising phase of systolic Ca^2+^ (see below). However, bridging the gap between measurements of Ca^2+^ spark sites, systolic Ca^2+^ synchronicity in living cells, and simultaneous or even sequential super-resolution imaging of the same RyR clusters is an ongoing challenge. It is worthy of consideration as to whether RyR expression, distribution, or function changes in cardiac disease states. Many studies have suggested previously that RyR opening is increased in disease (see below). Beyond these functional changes, a recent study by Li et al^35^ suggests that the distribution of RyRs also shifts in heart failure with higher densities of receptors being observed at cell ends where t-tubule density is decreased. Thus, to a first approximation at least the simulation studies noted previously seem to have some experimental basis although considerable further study in this area is still required.

Beyond the archetypal L-type Ca^2+^ channel-RyR dyad, consideration should also be given to other Ca^2+^ regulatory proteins that may be localized within the dyad. The limited studies that have systematically investigated the localization of NCX to the cardiac dyad give divergent results. For example, using confocal approaches (thus with limited resolving power below ≈200 nm), Scriven et al^36^ reported very low colocalization coefficients between NCX and RyRs in rat ventricle, whereas RyR and the L-type Ca^2+^ channel exhibited high colocalization. Thus, the authors concluded that NCX did not form part of the dyad in ventricular myocytes. In atrial myocytes, the same group found that some NCX was in the dyad but its degree of localization there was less than that of the L-type channel.^37^

Conversely, using immuno-gold labeling with electron microscopy methods, Thomas et al^38^ were able to identify a population of NCX within 100 nm of RyR clusters and concluded that this dyadic NCX had the potential to regulate Ca^2+^ fluxes within the dyad and influence systolic Ca^2+^. Consistent with this, a proportion of NCX colocalizing with RyRs has also been noted^39^ with similar implications for dyadic Ca^2+^ signaling being suggested.

The second protein of interest is SERCA. Available data on SERCA2 distribution in the heart is surprisingly sparse; however, confocal studies seem to suggest that a significant proportion of SERCA is localized to the z-line^40–42^ as well as enveloping the myofilaments.^43^ Thus, there is a possibility that both NCX and SERCA could be sufficiently close to the SR Ca^2+^ release sites as to modulate dyadic and thence cytosolic Ca^2+^. In the normal myocardium, systolic Ca^2+^ is tightly controlled, and while Ca^2+^ sparks may occur, these do not ordinarily form proarrhythmic Ca^2+^ waves. Subsequent sections will address in more detail how Ca^2+^ sparks may be self-terminating localized events; however, here we can posit that a dyadic population of SERCA and NCX may act as a firebreak and prevent these localized Ca^2+^ release events from activating adjacent RyR clusters and leading to triggering of Ca^2+^ waves. Indeed, there is evidence that SERCA activity can modulate the time course of Ca^2+^ sparks with increasing SERCA activity accelerating Ca^2+^ spark decay.^44^ Given this position, reductions in SERCA activity (via either reduced expression or hypophosphorylation of phospholamban^45,46^) in the diseased heart, especially if coupled to increased RyR density^35^ and thence an increased probability of Ca^2+^ spark occurrence,^32,33^ may impair the protective firebreak and facilitate the formation of Ca^2+^ waves and triggered activity.

## Ca Flux Balance

In the steady state, on each cardiac cycle, the amount of Ca entering the cell must equal that pumped out. If not, the cell would either gain or lose Ca. Imbalances between Ca entry and exit can only occur transiently and then result in changes of the amplitude of the Ca transient and thence contractility. One well-known example is the effect of changing frequency or pausing stimulation. If stimulation is stopped in ventricular muscle from most nonrodent species, Ca leaks out of the SR,^47^ SR content decreases, and therefore the first stimulus results in a small Ca transient and contraction.^48^ Because the Ca transient is small, less Ca is pumped out of the cell than enters and the cell is not in Ca flux balance. This results in an increase of SR Ca content until the Ca transient increases sufficiently that the Ca efflux now balances influx and the cell is back in a steady state. In the steady state, however, influx and efflux must be equal.^49,50^ The need for Ca flux balance applies not only to the surface membrane but also to organelles such as SR and mitochondria (see below).

## How Is Flux Balance Achieved?

**Figure 2.**
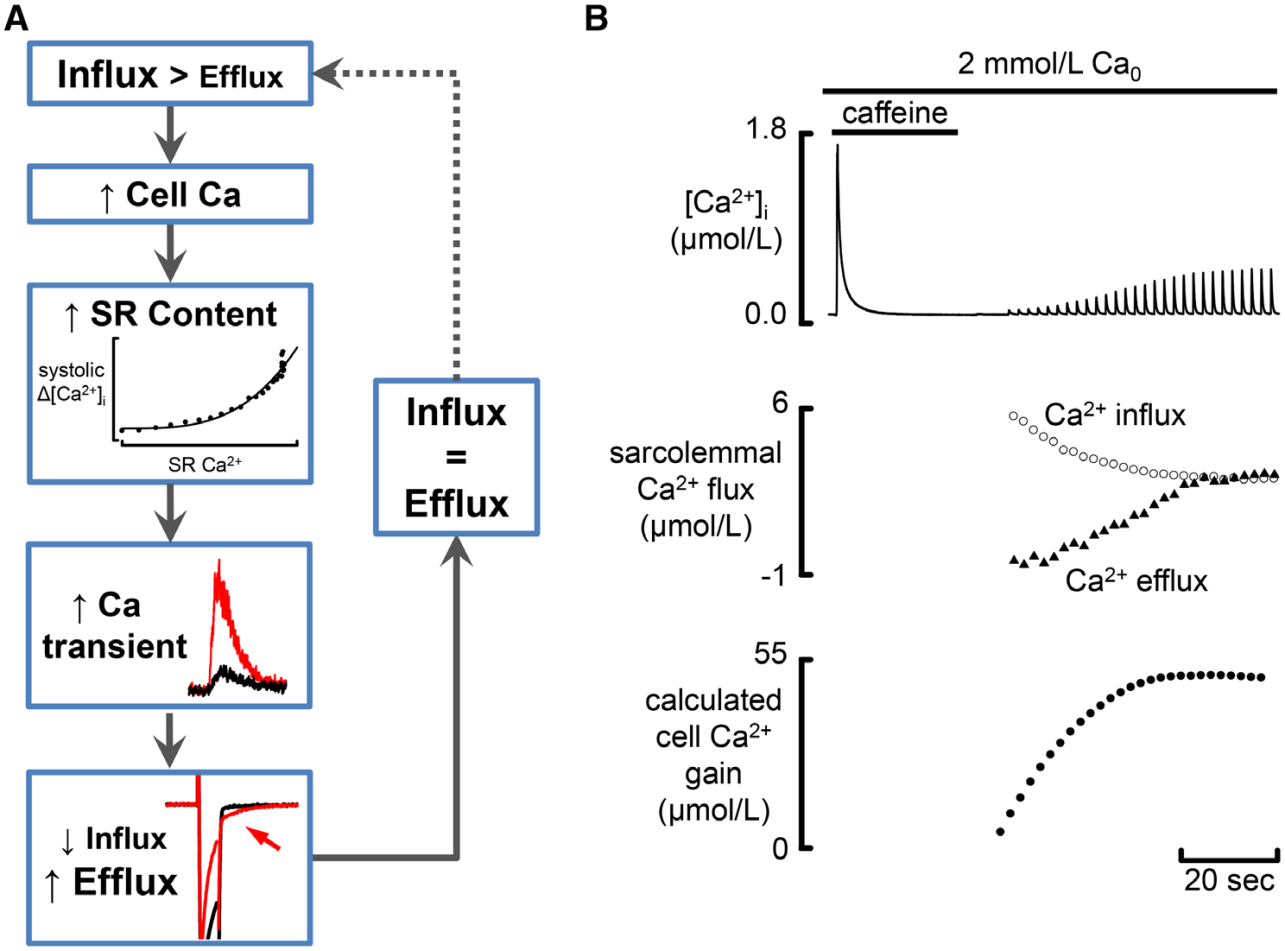
**Mechanisms producing calcium flux balance and controlling sarcoplasmic reticulum (SR) Ca content. A**, Flow diagram. This illustrates recovery from a situation where influx is greater than efflux. Boxes show (from **top** to **bottom**): increase of cell Ca content leading to an increase of SR Ca; increase of the amplitude of the Ca transient (red). The **bottom** box shows membrane current records in response to a depolarization. The red traces show that increase in size of the Ca transient leads to faster inactivation of the L-type Ca current during the pulse and a larger sodium–calcium exchange current on repolarization (arrowed). **B**, Illustrative traces. These show (from **top** to **bottom**) [Ca^2+^]_i_; sarcolemmal fluxes; calculated cell (and SR) Ca gain. At the start of the record, 10 mmol/L caffeine was applied to empty the SR. After removing caffeine, stimulation was commenced. Note that the recovery of the amplitude of the Ca transient is accompanied by a decrease of Ca influx and increase of efflux. Reprinted from Trafford et al^84^ with permission of the publisher. Copyright ©2001, American Heart Association, Inc.

This results from the negative feedback scheme of Figure 2A, which illustrates how the cell responds to a situation in which Ca influx is greater than efflux. (1) The imbalance of fluxes increases cell and therefore SR Ca. (2) Ca release is a steep function of SR Ca content^51,52^ and therefore the amplitude of the Ca transient increases. (3) Increasing the amplitude of the Ca transient increases Ca efflux and decreases Ca entry into the cell. This is because of a combination of 2 factors^52^: (1) Ca efflux on NCX is increased by increasing [Ca^2+^]_i_^53^ and (2) increased [Ca^2+^]_i_ increases Ca-dependent inactivation of the L-type Ca current.^54^ (3) This net loss of Ca from the cell decreases SR Ca. These events continue until Ca influx and efflux are equal. A good example of this mechanism in operation is provided by Figure 2B, which shows what happens when the SR has been emptied by exposure to 10 mmol/L caffeine. When stimulation is recommenced, the Ca transient is small because of the low SR Ca content. Consequently, Ca influx is much larger than efflux and the SR Ca content increases. This leads to an increase in the amplitude of the Ca transient until influx and efflux return to balance.

Work on Ca cycling often takes insufficient notice of the flux balance condition. As discussed below, it is essential that postulated mechanisms and explanations are tested to ensure that they are compatible with the requirement for Ca efflux to equal influx such that steady state conditions can prevail.

## Examples of the Effects of Flux Balance on Ca Handling

### Effects of Altering Sarcolemmal Ca Fluxes

A change of Ca entry must be balanced by a change of [Ca^2+^]_i_. Starting off from a steady state, an increase in the L-type Ca current will mean that influx is greater than efflux. This will increase the amount of Ca in the cell and SR until the resulting increase of the amplitude of the Ca transient increases efflux to a level that restores flux balance. What magnitude increase of the Ca transient is required to bring the cell back into flux balance? At first sight, this appears to be an intractable problem as at least 2 factors have to be considered. (1) An increase of Ca entry will increase the number of RyRs that open, and the size of this effect will depend on the relationship between dyadic [Ca^2+^] and RyR opening. (2) Increased Ca entry might be expected to increase SR Ca content (but see below), and therefore the amount of Ca released from the SR through each RyR that opens. The analysis is, however, simplified by the requirement for flux balance. Specifically, the increase of Ca entry must be balanced by an equal increase of Ca efflux. Assuming that diastolic [Ca^2+^]_i_ does not change, the increased efflux will be provided by an increase of the amplitude of the Ca transient. This is irrespective of the underlying mechanisms. If we make the reasonable assumption that the rate of NCX is proportional to [Ca^2+^]_i_,^53^ then the amplitude of the Ca transient must increase by the same proportion as the Ca entry. Likewise, slowing sarcolemmal extrusion by NCX increases the amplitude of the Ca transient to a level that restores the efflux to balance the influx.^55^

### Effects of Altering Intracellular Mechanisms

What happens if Ca transporters across intracellular membranes such as SR or mitochondria are affected? The simple answer is that if the Ca influx into the cell is unchanged, then the Ca efflux must be unaffected. This either means that the amplitude and kinetics of the systolic Ca transient are unaffected or that a change of amplitude is exactly compensated by one of time course such that efflux is unaffected (see below for consideration of mitochondrial function).

**Figure 3.**
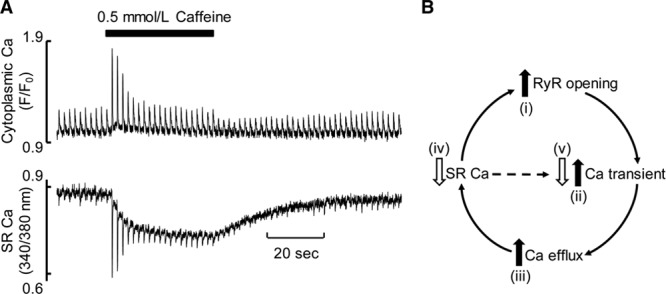
**Effects of potentiating ryanodine receptor (RyR) opening. A**, Records show measurements of (**top**) cytoplasmic and (**bottom**) sarcoplasmic reticulum (SR) [Ca^2+^]. Caffeine (0.5 mmol/L) was added for the period shown. Reprinted from Greensmith et al^58^ with permission. Copyright ©2014, The Authors. Published by Oxford University Press on behalf of the European Society of Cardiology. **B**, Flow diagram of events underlying changes in **A**. (i) The increase of RyR opening increases the amplitude of the Ca transient (ii) leading to increased Ca efflux (iii) and a decrease of SR Ca content (iv) which returns the amplitude of the Ca transient to control levels (v). Reprinted from Eisner^183^ with permission. Copyright ©2014, The Author. Published by the Physiological Society.

A striking example is provided by considering the effects of changing the open probability of the RyR. Adding submillimolar concentrations of caffeine potentiates the opening of the RyR (without affecting Ca^2+^ entry via *I*_Ca_), increasing the amplitude of the systolic Ca transient. After a few beats, however, the amplitude of the Ca transient in caffeine is identical to that in control^56,57^ (Figure 3A). The explanation of this result is that potentiation of RyR opening initially increases the amplitude of the Ca transient making efflux greater than influx so the cell is no longer in a steady state. The SR therefore loses Ca, decreasing the amplitude of the Ca transient until a new steady state (influx=efflux) is reached, with a decreased SR Ca content offsetting the potentiation of the RyR produced by caffeine. This occurs when the amplitude of the Ca transient returns to the control (pre-caffeine) level (Figure 3B). The underlying decrease of SR Ca, responsible for the decline of the Ca transient amplitude to the control level, has been measured directly using a fluorescent indicator in the SR^58^ (Figure 3A).

These arguments were originally made with respect to the effects of low concentrations of caffeine, but similar effects are seen when the RyR is potentiated with BDM (2,3-butanedione monoxime).^59^ Likewise, decreasing RyR opening with tetracaine^60^ or decreased pH^61^ produces a transient decrease of contraction or the Ca transient. This analysis can be generalized to other mechanisms that alter RyR opening. For example, phosphorylation of the RyR increases its open probability and this has been suggested to contribute to the positive inotropic effects of β-adrenergic stimulation.^62^ This conclusion has been criticized on other grounds,^63^ but, in the context of flux balance, seems implausible as any increase of the Ca transient would make efflux exceed influx, resulting in the Ca transient returning to the control level. As discussed elsewhere,^64^ the positive inotropic effects of β-adrenergic stimulation can be explained by the well-established effects to (1) increase Ca influx via the L-type current^65^ and (2) increase SR content by phosphorylating PLN and thereby stimulating SERCA.^66^

What are the effects of altering SERCA activity? An increase (in the absence of effects on sarcolemmal fluxes) will increase SR Ca thereby increasing the amount of Ca released and thence the Ca efflux from the cell. This effect alone would make efflux greater than influx, thus violating flux balance, but it will, however, be offset by the fact that increased SERCA activity increases the rate of decay of the Ca transient thereby allowing less time for Ca extrusion. In the steady state, the increase in the amplitude of the Ca transient must exactly balance the acceleration of its decay so that efflux is unaltered. Put another way, the fraction by which the amplitude of the Ca transient increases is determined by the acceleration of SERCA.

This section has given some examples of the importance of flux balance considerations to understanding changes of contractility. The consequences of flux balance will also be referred to in subsequent sections.

## Ca Buffering

The increase of free Ca ([Ca^2+^]_i_) during systole is of the order of 1 µmol/L. Ca is, however, strongly buffered; for every free Ca^2+^ ion, ≈100 to 200 are bound to buffers,^67,68^ meaning that the total increase of cytoplasmic Ca is of the order of 100 to 200 µmol/L. The major Ca buffers are troponin and SERCA.^3^ Buffering by SERCA requires some discussion as it is generally thought of only as a Ca pump. Although this is, indeed, the case, the initial binding of Ca by SERCA contributes significantly to buffering.^69^ This buffering increases the fluxes of Ca required to produce a given change of [Ca^2+^]_i._ Similarly, buffering decreases the rate constant of decay of cytoplasmic Ca. The effects of Ca buffers will also depend on the kinetics with which they bind and unbind Ca. A very fast buffer will decrease the amplitude of the Ca transient and slow its decay, as a given Ca flux produces a smaller change of [Ca^2+^]_i_. This will result in decreased systolic and increased diastolic [Ca^2+^]_i_, an effect that can be a problem experimentally when excessive concentrations of Ca indicators (buffers) are used.^70^ If, however, the buffer binds Ca more slowly, the initial amplitude of the Ca transient will be large but will decay with a rate given by the binding of Ca to the buffer.^71^ In skeletal muscle, parvalbumin has similar effects. Binding of Ca^2+^ is slow because Mg^2+^ must first dissociate thereby allowing [Ca^2+^]_i_ to rise (for review see^72^) resulting in a buffer that is most active during diastole. It has been suggested that incorporating such a buffer into the heart would, therefore, preserve the systolic rise of [Ca^2+^]_i_ (when buffering is weak) but lower diastolic [Ca^2+^]_i_ (when it is strong). In support of this, the incorporation of parvalbumin protects against increased diastolic [Ca^2+^]_i_ in the Dahl salt-sensitive rat.^73^ Finally, it may well be that buffering is not uniform throughout the cytoplasm. For example, the dyadic space contains no myofibrils, and therefore troponin will not contribute to the buffering here.

## Factors That Affect Ca Buffering

Ca buffering depends on [Ca^2+^]_i_; buffer power is greatest at low levels of [Ca^2+^]_i_ and decreases at higher levels as the buffers tend to saturate. This may play a role in determining the rate of relaxation of the systolic Ca transient. For example, the reduced buffering at the highest [Ca^2+^]_i_ will mean that a given rate of pumping by SERCA will reduce [Ca^2+^]_i_ more quickly at the start of the decay of the Ca transient resulting in a biphasic decay of [Ca^2+^]_i_.^74^ However, other factors affect buffer power, even at a constant [Ca^2+^]_i._ In some cases, this is genetic. Familial hypertrophic cardiomyopathy is caused by mutations in myofilament proteins including thin filament proteins such as troponin and tropomyosin. Many of these mutations result in an increase of Ca binding to troponin^75^ and thence increased Ca buffering.^76^ This increased buffering was correlated with an increase of both diastolic [Ca^2+^]_i_ and the probability of triggered Ca waves.^76^ Ca buffering can also be modified acutely, by phosphorylation. As regards the 2 major buffers, phosphorylation of troponin decreases its affinity for binding Ca whereas phosphorylation of PLN increases the affinity of SERCA for Ca and presumably its buffering. The expected changes of buffering produced by β-adrenergic stimulation have been demonstrated experimentally. In cells from wild-type mice, β-adrenergic stimulation has no effect on buffering power as the decrease in buffering by troponin is compensated for by the increase because of SERCA.^77^ The individual effects could, however, be revealed in cells from animals in which either the regulation of troponin or SERCA by β-adrenergic stimulation was prevented.^77^ A final question on buffering is whether it is affected by disease. No changes in buffering were observed in ventricular myocytes when heart failure was induced.^78^ In atrial fibrillation, however, an increase of buffering power has been suggested to decrease propagation of the Ca transient into the interior of the atrial myocyte.^79^ Given the extensive changes in SERCA expression found in many models of heart failure,^80,81^ it is perhaps surprising that changes of buffering have not been reported more generally and this area would warrant study.

## Regulation of SR Ca Content

Direct measurements of intra-SR free Ca concentration provide values of 1 to 1.5 mmol/L at the end of diastole with the concentration decreasing by 50% to 75% during contraction.^82^ As mentioned above, a major factor controlling the amount of Ca released from the SR and thereby the amplitude of the Ca transient is the SR Ca content. It is, therefore, important to understand the regulation of SR Ca content. In brief, SR content is determined by the balance between uptake of Ca into the SR (by SERCA) and efflux (through the RyR). In turn, these fluxes depend, not only on the properties of SERCA and RyR but also on the Ca concentration in the cytoplasm and SR. The feedback mechanism shown in Figure 2 to explain cellular Ca flux balance also serves to explain regulation of SR Ca content (see^83^ for review). SR content will change until the Ca transient is the exact amplitude required to produce a Ca efflux that balances the influx. If the SR content is below this value, then efflux will be less than influx and the cell (and SR) Ca content will increase. The steady state value of SR Ca content reached will be altered by changing the expression or properties of any of the Ca-handling proteins. As reviewed above, increasing SERCA activity or decreasing RyR opening will increase SR content and increasing NCX will decrease it. The most complicated factor is the L-type Ca current. At first sight, one might think that an increase of the L-type Ca current will load the cell with calcium and thence increase SR content. Experimentally, however, even a large increase of the L-type current has little effect on SR content, and a decrease increases content.^84^ Indeed, in a sheep model of heart failure, a decrease of L-type current was suggested to cause the observed increase of atrial SR content.^85^ This is because the L-type current plays 2 roles in Ca cycling. The peak amplitude determines the triggering of Ca release from the SR so an increase of current will decrease SR content, whereas the rest of the current loads the cell and SR with Ca.^86^ The net effect on SR Ca content of a change of L-type current will depend on the relative strength of the 2 opposing effects on the SR. Under basal conditions, it appears that these are matched so that there is little effect on SR content.^84^ This may be physiologically useful as it means that an increase of L-type Ca current will produce an immediate increase in the amplitude of the Ca transient without the delay produced by the need to increase SR content.^84,87^

## Need for Adequate Measurement of SR Ca Content

Many experimental studies involve addressing whether a change in the amplitude of the systolic Ca transient results from one of SR Ca content. Undoubtedly, the best way to measure SR Ca is to use a Ca-sensitive indicator in the SR.^82,88^ Such indicators, however, do not seem to work in all tissues.^58^ There is also a problem with saturation of the indicator at the high [Ca^2+^] in the SR. A simple way to measure SR content is to release all the Ca into the cytoplasm by applying 10 mmol/L caffeine and measuring the amplitude of the resulting increase of [Ca^2+^]_i._ The problem here is that the level of [Ca^2+^]_i_ at the peak of the caffeine response is close to those that saturate commonly used Ca indicators thereby reducing the sensitivity of the measurement, an issue that is exacerbated by the steepness of the dependence of Ca transient amplitude on SR Ca. We suggest that, before rejecting the hypothesis that a change of Ca transient amplitude results from one of SR Ca content, it is essential to see how large a change of content would be required to explain the effect and whether the measurement has sufficient sensitivity to detect it. Problems of saturation of the indicator can be mitigated by using a lower affinity calcium indicator.^89^ Alternatively, if the experiment can be performed under voltage clamp, then a more accurate estimate of SR Ca content can be obtained by measuring the integral of the NCX current activated by the caffeine-evoked increase of [Ca^2+^]_i_.^90^

## Calcium Release From the SR

Before considering mechanisms that may control Ca release from the SR, it is important to remember that the RyR does not sit in isolation in the SR membrane but, rather, forms a complex with triadin, junctin, and CSQ (calsequestrin).^91^ CSQ is the major Ca buffer in the SR but has been suggested to have other effects because it, in addition to triadin and junctin, is required to make RyR open probability respond to luminal Ca, at least in bilayer studies.^92^

The phenomenon of calcium-induced calcium release has been appreciated for ≈50 years.^93^ A major concern for much of this time was the issue of how it was regulated. As originally described, calcium-induced calcium release is a positive feedback system in which one might expect the Ca released from the SR to trigger further release of Ca until the SR is empty. This contrasts with the observation that Ca release is graded with the amplitude of the L-type Ca current^94,95^ and, indeed, the SR only releases ≈50% of its Ca during the Ca transient.^96^ The resolution of this paradox came from both modeling^97^ and experimental work showing that, under normal conditions, Ca release from one release site of the SR remains localized and does not activate other release sites. In other words Ca release is controlled locally. Under resting conditions, localized releases of Ca from individual clusters of RyRs are seen as Ca sparks.^98^ Depolarization of the surface membrane activates more and more L-type Ca channels resulting in an increasing number of sparks until spatially uniform Ca release is observed.^99^

As mentioned above, Ca release from the SR is a steep function of SR Ca content.^51,52^ This steep dependence is functionally important, not only does it contribute to regulation of flux balance but also it provides a mechanism to regulate contractility. The steep dependence is a consequence of several factors including the fact that an increase of SR Ca content (1) increases the driving force for Ca release through open RyRs and (2) increases the number of open RyRs. As noted below, the latter effect may not be directly because of SR Ca but, rather, secondary to Ca release from the SR. Too steep a dependence of Ca release on SR content may result in instability of Ca release resulting in such phenomena as alternans.^100,101^ Finally, when SR Ca content exceeds a certain threshold level, the local regulation of Ca release breaks down, propagating Ca waves are observed^102^ (see Calcium Leak section of this article).

Although the discovery of the spark resolved the question of how Ca release could be graded rather than all or none, it raised another difficulty; how does release terminate so that the SR can refill with Ca? The problem is that Ca released from the SR would be expected to continue to activate RyRs in the same cluster thereby maintaining Ca release. Various explanations have been considered. (1) The release process may inactivate, even in the presence of constant activating Ca.^103,104^ A related phenomenon, known as adaptation (where the RyR can still be opened but requires a larger stimulus), has also been identified.^105,106^ The rate of this inactivation/adaptation may, however, be too slow to be the only factor involved in terminating Ca release (see^107^ for review). (2) An alternative, termed stochastic attrition, depends on there being a probability that all the RyRs in a cluster close by chance such that the Ca outside the SR will fall to levels too low to open RyRs. Although this would work well if there were only a small number (up to ≈15) of RyRs in a cluster, it is less plausible given experimental data showing that there can be >100 RyRs per cluster.^10,30,31^ (3) Another explanation depends on changes of lumenal Ca. The fact that, even at the end of the release, the SR still contains 25% to 50% of its Ca content^82^ is inconsistent with the idea that a simple effect on driving force accounts for the turn off of release. The decrease of SR Ca will also decrease the frequency of RyR opening,^108,109^ but this effect, alone, is probably not strong enough to terminate release. A variety of modifications has been made to try to account for termination. One, the sticky cluster model^110^ is based on the observation that the opening of 2 RyRs can be coupled such that they open and close together.^111^ This makes it easier for stochastic attrition to occur and together with SR depletion could account for spark termination. The difficulty is that coupled gating is not observed in most bilayer studies. More recent studies have suggested that luminal Ca may still be the controlling factor but via an indirect mechanism involving effects on cytoplasmic activation of the RyR. Evidence that luminal concentration per se is not the important factor is supported by the fact that large organic cations that decrease Ca flux through the RyR increase SR content while decreasing spark frequency.^112^ This is consistent with a model in which initially 1 RyR opens; the resulting increase of [Ca^2+^]_i_ leads to or induces the opening of adjacent RyRs and thence a Ca spark. The release of Ca decreases luminal [Ca^2+^] in the junctional SR, decreasing release to a level where [Ca^2+^]_i_ is sufficiently low that all RyRs close. This model has been called induction decay^113,114^ and pernicious attrition^115^ (see also^32^). In these models, the start and end of Ca release depend on (1) the sensitivity of the single-channel current to luminal Ca and (2) the activation by cytoplasmic Ca. Consistent with this model, a recent study has demonstrated that increasing cytoplasmic Ca buffering (thereby impeding the activation of neighboring RyRs) makes sparks terminate at an elevated SR Ca content.^116^

A phenomenon that is related to that of termination of release is that after 1 stimulated release, there is a refractory period before another full release can occur. Early attempts to investigate this experimentally suffered from the fact that the triggering L-type Ca current itself requires time to recover from inactivation.^117^ When this issue was overcome using photolysis of caged Ca to trigger Ca release, it was found that Ca release recovered with a time constant of ≈300 ms.^118^ This slow recovery was absent if only a small region of SR was stimulated (using 2 photon photolysis) leading to the conclusion that the refractoriness was because of depletion of SR Ca and presumably would involve the mechanisms described above. Further support for a role for Ca depletion was provided by the observation that incorporation of Ca buffers into the SR decreased apparent refractoriness.^119^ Simultaneous measurements of SR and cytoplasmic Ca suggest, however, that refractoriness may result from something in addition to SR Ca. After the first stimulus, SR Ca content (as measured with an intra-SR indicator) recovered fully before maximal Ca release could be obtained.^120^ This dissociation is partly explained by the fact that the indicator is tending to saturation, but this cannot explain everything. Another explanation might be that the SR Ca concentration at the release sites recovers more slowly than that in the bulk SR. Finally, it is possible that there is a genuine Ca-independent refractoriness of the RyR.

## Calcium Leak

The emphasis above has been on the release of Ca from the RyR in response to triggering by the L-type Ca current. However, given that the RyR has a finite open probability even at diastolic [Ca^2+^]_i_, Ca will leak out of the SR. Early evidence for a leak came from the phenomenon of rest decay where, after a pause in stimulation, the first contraction is smaller than the steady state and this is associated with a decrease in total cell^121^ and SR^47^ Ca. Some, but not all, of this leak occurs via Ca sparks. Work on rabbit ventricular myocytes that had been skinned (ie, the surface membrane was removed) found that inhibiting SERCA with thapsigargin decreased both SR Ca content and the frequency of Ca sparks.^122^ A point was then reached when, although SR Ca content continued to decrease, no sparks were observed. The decrease of Ca spark frequency as SR Ca falls is to be expected from the effect of SR Ca on RyR opening.^108^ Most of the spark-independent decrease of SR Ca was inhibited by blockers of the RyR such as tetracaine, indicating that it is because of the unsynchronized opening of individual RyRs. Modeling suggests that stochastic considerations determine which RyR opening result in a Ca spark.^123^ Finally, SR Ca continued to decrease, even when the RyRs were inhibited indicating an additional mechanism for leak efflux from the SR.

In the experiments described above, changes of Ca leak resulted from those of SR Ca content. However, Ca leak is also a function of the properties of the RyR itself and associated proteins. SR Ca leak is elevated by single amino acid mutations such as those occurring in catecholaminergic polymorphic ventricular tachycardia in either the RyR^124^ or CSQ.^125^ Leak is also increased in heart failure as was originally shown by measuring the open probability of RyRs incorporated into bilayers. Those from dogs with heart failure had a higher open probability than from control animals, an effect that was suggested to result from excessive phosphorylation of the RyR leading to the dissociation of the regulatory protein FKBP12.6^126^. Although there is general agreement of increased SR Ca leak in heart failure, the precise mechanism remains controversial. The concentration of FKBP12.6 in the ventricular myocyte is too low to bind to more than a small minority of RyRs and phosphorylation by PKA (protein kinase A) has no effect on binding.^127^ In contrast, there is substantial evidence for a major role for Ca/calmodulin-dependent protein kinase II—dependent phosphorylation^128,129^ as well as for oxidation either directly affecting the RyR^130^ or indirectly via Ca/calmodulin-dependent protein kinase II of the RyR.^131^ For a recent review of this area, see the study by Bers.^132^

## Consequences of Ca Leak

A major effect of Ca leak is to decrease the Ca content of the SR and thence the amplitude of the Ca transient. In this context, an important issue concerns the properties of the leak. Evidence from the Gyorke group using bilayer studies has found that in heart failure, there is an apparent sensitization of the RyR to activation by luminal Ca.^133^ This contrasts with a previous study, suggesting that heart failure locked the RyR in a subconducting state.^126^ The difference is significant for the effects of leak on the systolic Ca transient. If the leak results from a mechanism that sensitizes the RyR, then (as for the effects of low concentrations of caffeine mentioned above) the sensitization of the RyR will initially compensate for the decrease of SR Ca content and no effect will be seen on the amplitude of the Ca transient. As the leak increases, the SR Ca content falls to such a low level that even the release of 100% cannot sustain a normal-sized Ca transient and the amplitude of the Ca transient declines^134^ and efflux is maintained by prolongation of decay. In contrast, if the leak does not result from sensitization of the RyR, then the amplitude of the Ca transient will decline in parallel with SR content. An analogy is provided by comparing the effects of caffeine (sensitizing-) with those of ryanodine (nonsensitizing-) leak. In ryanodine, SR Ca and Ca transient amplitude decay together, whereas, in caffeine, the amplitude of the Ca transient is preserved at low levels of leak.^135,136^

Increased Ca leak is arrhythmogenic as a result of the occurrence of intracellular Ca waves that occur when the Ca spark frequency and flux rises so that Ca spreads beyond the original site and activates others.^137^ The waves that activate NCX,^138^ giving rise to arrhythmogenic delayed afterdepolarizations^139,140^ and resulting arrhythmias, were originally described for situations where the SR Ca content was elevated to above a threshold level^102^ (sometimes referred to as store overload–induced Ca release^141^) but also occur when the RyRs are modified as in catecholaminergic polymorphic ventricular tachycardia and heart failure (see^142,143^ for reviews). The occurrence of these waves also relates to Ca flux balance as they activate a component of Ca efflux from the cell in addition to that produced by the systolic Ca transient. For example, when a cell that has an increased Ca load (eg, because of β-adrenergic stimulation) is treated with caffeine, Ca waves develop. To maintain flux balance, there is a compensatory decrease of the amplitude of the systolic Ca transient.^144^ Flux balance considerations also determine whether making the RyR leaky, either in a natural disease such as catecholaminergic polymorphic ventricular tachycardia or experimentally with caffeine, results in Ca waves. If the Ca influx into the cell on each beat is below a certain level, then making the RyR leaky will not produce waves. Only if there is sufficient influx to balance the extra (wave-associated) Ca efflux, will waves result.^64,144,145^

Finally, Ca leak occurring during the decay of the Ca transient will slow its decay as it adds an additional flux to compete with SERCA.^146^ This is observed with concentrations of caffeine above ≈1 mmol/L.^135,147^ The increased leak can also result in a biphasic decay of the Ca transient, an effect attributed to the flux through the open RyRs being low at the start of the decay of Ca transient and therefore not competing greatly with SERCA. As the SR refills, then the leak efflux increases and the rate constant of decay slows.^136^

## Mitochondria and Calcium

The mitochondrial inner membrane contains a calcium channel, the mitochondrial Ca uniporter (MCU), identified a few years ago^148,149^ (see^150^ for recent review). Ca entry into the mitochondrion is driven largely by the inside-mitochondria negative membrane potential. Mitochondria are often located adjacent to the junctional SR, and it is therefore been suggested that Ca release will elevate local Ca to high levels resulting in a large influx.^151^ Such an influx is important for mitochondrial function as many of the mitochondrial enzymes are activated by a rise of matrix calcium concentration leading to increased supply of ATP when demand, because of increased contraction and [Ca^2+^]_i_, is increased. As far as understanding E–C coupling is concerned, it is important to know whether on each beat a significant amount of the Ca released from the SR enters the mitochondria. Flux balance conditions require that, in the steady state, if Ca enters the mitochondria on 1 beat, exactly the same amount must leave before the next and therefore a transient change of mitochondrial [Ca^2+^] would be expected. Although some early studies reported beat-to-beat changes of mitochondrial Ca, others did not (see^152,153^ for reviews). Earlier studies, particularly in adult myocytes, suffered from problems of specifically measuring mitochondrial Ca. A more recent study, using a mitochondrially targeted Ca sensor, found beat-to-beat Ca transients that were larger at regions of mitochondria near to the SR^151^. It should, however, be noted that the mitochondrial Ca transients decayed with a time constant of ≈5 seconds. This slow rate is probably a function of the low level of Ca efflux through the mitochondrial Na–Ca exchange. When stimulation rate was increased from 0.1 to 0.5 Hz, the beat-to-beat mitochondrial Ca transients disappeared and were replaced by a virtually tonic increase of mitochondrial Ca concentration. Even allowing for the fact that the experiments were performed at room temperature and therefore the mitochondrial Ca transients may decay more quickly at 37°C, this result makes it less likely that mitochondrial Ca transients are of physiological importance. This study^151^ also investigated the question as to whether any flux of Ca into the mitochondria affects the cytoplasmic Ca transient. This was done by comparing the cytoplasmic Ca transient in control cells with those incubated with the MCU inhibitor, Ru360. The amplitude of the cytoplasmic Ca transient was identical in both groups, suggesting that the total flux of Ca into the mitochondria is small when compared with that released from the SR and therefore makes little contribution to relaxation.^151^ This is consistent with a previous study, showing that mitochondria have little effect on Ca removal from the cytoplasm.^154,155^ Work on neonatal myocytes also found mitochondrial Ca transients that were abolished by knocking down the MCU with siRNA.^156^ However, knockdown of MCU increased the amplitude of the cytoplasmic Ca transient by ≈50% to 60% leading to the conclusion that there is a significant beat-to-beat flux of Ca into the mitochondria that buffers the cytoplasmic Ca transient. As well as being at odds with the work on adult cells, this result is difficult to interpret in the context of flux balance considerations. Simply increasing the amplitude of the Ca transient (as occurs when MCU is knocked down) would be expected to increase the efflux of Ca from the cell thereby making it greater than the influx, a situation that cannot persist in the steady state. If Ca is taken up into the mitochondria, then it must be released between beats, an effect that should slow the rate of decay of [Ca^2+^]_i_ and elevate diastolic [Ca^2+^]_i._ Knockdown of the MCU might therefore be expected to accelerate the decay of the Ca transient.^156^ This would compensate for the increased amplitude and thereby restore Ca efflux to control levels, achieving flux balance. No effect on the rate constant of decay was, however, reported, thus leaving open the question as to how the result can be squared with flux balance. Finally, for completeness, it should be noted that, although the MCU is required for rapid regulation of mitochondrial Ca,^157^ mitochondrial Ca can still change, albeit more slowly when this has been deleted,^158^ suggesting the existence of another mechanism for Ca to enter the mitochondria.

## Control of Diastolic Ca

As mentioned above, the ability of the heart to pump blood depends as much on a low diastolic [Ca^2+^]_i_ as on the systolic elevation. Indeed diastolic heart failure is a major cause of morbidity with many patients having no apparent impairment of systolic function (heart failure with preserved ejection fraction).^159^ Diastolic heart failure may well involve many factors other than [Ca^2+^]_i_ regulation, with fibrosis being a significant factor.^160^ That said, it is clearly important to understand the regulation of diastolic [Ca^2+^]_i_. As will become apparent below, understanding of the control of diastolic Ca lags behind that of systolic Ca.

## Resting Ca

The heart beats continuously. Nevertheless, as a first step in understanding the mechanisms involved in the control of diastolic Ca, a considerable amount of work has studied quiescent cardiac preparations. In the absence of stimulation, in the steady state, there must be no net flux across the membranes of the SR and other organelles and the level of resting [Ca^2+^]_i_ is therefore controlled entirely by the surface membrane.^161,162^ Early studies showed that resting [Ca^2+^]_i_, either measured directly or using resting force as a surrogate, was very sensitive to the sodium gradient with NCX being the main mechanism responsible for pumping Ca out of the cell.^163,164^ The surface membrane also contains a plasma membrane Ca-ATPase (PMCA), which should also contribute to Ca efflux. In rat ventricular myocytes, PMCA has been estimated to make a contribution equal to between 7%^165^ and 25%^166^ of that produced by NCX. It is unclear what is responsible for the 3-fold range of these estimates. Given that the level of resting [Ca^2+^]_i_ represents the balance between Ca influx and extrusion, it is important to identify the Ca entry mechanism in a quiescent cell. In rat ventricular myocytes, we estimated a background Ca entry of the order of ≈2 to 5 µmol/L per second.^167^ This compares with that of the order of 4 µmol/L for the entry through the L-type current,^84^ which at a typical heart rate of 6 Hz corresponds to 24 µmol/L per second, a considerably larger value. The identity of this Ca entry is unclear. At a normal resting potential, the open probability of the L-type channel is very low and therefore unlikely to make a major contribution. When both the L-type Ca channel and NCX were inhibited, this background entry mechanism was revealed by the decrease of [Ca^2+^]_i_ on maintained depolarization, an effect attributed to a decreased driving force.^168^ A major limitation of the analysis was the lack of a specific inhibitor with only gadolinium having a marked effect.

Work using the HL-1 cell line found a store-operated Ca entry (SOCE). Interestingly, inhibiting this entry mechanism also decreased the resting level of [Ca^2+^]_i_,^169^ suggesting that SOCE may contribute to resting Ca in unstimulated cells. Such SOCE has been identified in many cell types. Briefly, a decrease of endoplasmic reticulum Ca results in the opening of surface membrane channels leading to a refilling of the endoplasmic reticulum with Ca (see^170^ for review). The mechanism of this involves an endoplasmic reticulum Ca sensor (STIM1 [stromal interaction molecule 1]) which, when endoplasmic reticulum Ca is decreased, interacts with the surface membrane channel Orai.^171^ This mechanism is best characterized in nonexcitable cells, where it may be the major route for Ca entry into the cell, but is much less well characterized in cardiac myocytes. Early evidence for SOCE in the heart was obtained in neonatal cells,^172^ and the mechanism was reported not to exist in adult cells.^173^ Although some subsequent studies have revealed SOCE in adult myocytes,^174,175^ a recent study could not find it.^176^ The general consensus is that the mechanism is much more evident in the developing heart (see^177^ for review). It may also be important for the development of cardiac hypertrophy^178,179^ with overexpression of STIM1 leading to cardiomyopathy.^180^ A further complication in this field is the report that STIM1 increases SR Ca by activating SERCA secondary to interacting with PLN.^176^

## Diastolic Ca

**Figure 4.**
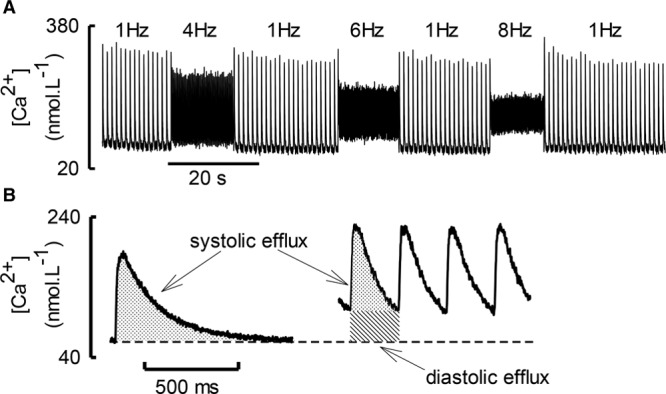
**Effects of stimulation rate on diastolic and systolic [Ca**^**2+**^**]**_**i**_**. A**, Original traces showing the effects of stimulation at the rates indicated. **B**, Diagrammatic representation of systolic efflux. At 1 Hz (**left**) efflux will be activated by the systolic rise of [Ca^2+^]_i_ (dotted area). At 4 Hz (**right**), systolic efflux has decreased (dotted) whereas diastolic has increased (diagonal lines). Reprinted from Dibb et al^181^ with permission. Copyright ©2007, The Authors. Published by the Physiological Society.

When the heart beats, the level of diastolic [Ca^2+^]_i_ is determined by a combination of sarcolemmal and SR fluxes. The beating cell provides an interesting illustration of the effects of flux balance. In much previous work,^57^ the low rate of stimulation results in diastolic [Ca^2+^]_i_ being constant at the resting level and flux balance is determined by systolic fluxes. When the cell is stimulated rapidly, then both systolic and diastolic levels of [Ca^2+^]_i_.are important. A good example is provided by the effects of increasing stimulation rate. Figure 4 shows that increasing rate decreases the amplitude of the systolic Ca transient.^181^ This is, in part, because of the decrease of the amplitude of the L-type Ca current.^182^ This is accompanied by an increase of diastolic [Ca^2+^]_i_ because, at higher rates, the previous Ca transient has not had time to decay before the next stimulus. However, the increased level of diastolic [Ca^2+^]_i_ will also increase efflux of Ca from the cell. This will compensate for the fact that the shorter and smaller Ca transient produces less efflux from the cell during systole.

## Conclusions

The work reviewed in this article illustrates the enormous progress that has been made in understanding calcium signaling in the heart. The next few years should see further rapid advances, helped to no small extent by technological advances in areas such as imaging. A major area that should develop greatly is that of the control of diastolic Ca.

## Sources of Funding

This work was supported by the British Heart Foundation (grant number: CH/2000004/12801).

## Disclosures

None.
